# Gray Hairs

**Published:** 1893-01

**Authors:** 


### GRAY HAIRS.

The fact that some persons begin to show gray hairs while in their twenties does not indicate a premature decay of the constitution. It is purely a local phenomenon, and often co-exists with great physical vigor. A medical journal says : Many feeble persons, and others who have suffered extremely, both mentally and physically, do not blanch a hair until past middle life ; while others, without assignable cause, lose their capillary coloring matter rapidly when about forty years of age.

Race has a marked influence. The traveler, Dr. Orbigny, says that in many years he spent in South America he never saw a bald Indian, and scarcely ever a gray headed one. The negroes turn more slowly than the whites.

In this country sex appears to make little difference. Men and women grow gray about the same period of life. In men the hair and beard rarely change equally. The one is usually darker than the other for several years, but there seems no general rule as to which whitens the first.

The spot where grayness begins differs with the individual. The philosopher, Schopenhauer, began to turn gray on the temples, and complacently framed a theory that this indicates vigorous mental activity.

The correlation of gray hairs, as well as its causes, deserve more attentive study than they have received. Such a change is undoubtedly indicative of some deep seated physiological process ; but what this is we can only ascertain after more extensive observations than have yet been submitted to science.

